# Factors associated with appointment non‐attendance at a medical imaging department in regional Australia: a retrospective cohort analysis

**DOI:** 10.1002/jmrs.284

**Published:** 2018-05-27

**Authors:** Gordon T. W. Mander, Lorraine Reynolds, Aiden Cook, Marcella M. Kwan

**Affiliations:** ^1^ Toowoomba Hospital Darling Downs Hospital and Health Service Toowoomba Queensland Australia; ^2^ Rural Clinical School Faculty of Medicine The University of Queensland Toowoomba Queensland Australia

**Keywords:** Medical imaging, non‐attendance, service evaluation

## Abstract

**Introduction:**

Appointment non‐attendance contributes added cost to the healthcare sector through wasted resource allocations. Medical imaging departments commonly schedule appointments for most modalities; however, no study has quantified patient attendance rates in the Australian regional setting. This is despite evidence that regional, rural and remote Australians tend to demonstrate poorer health than metropolitan counterparts. This study aims to identify the factors that influence appointment non‐attendance at a teaching hospital in regional Australia.

**Methods:**

Categories restricted to age, gender, indigenous status, distance from investigation site, referral source and imaging modality were collected for all appointments (*N* = 13,458) referred to the medical imaging department in 2015. The likelihood of each of these factors correlating with a patient not attending a scheduled appointment was calculated using the chi‐squared analysis and binary logistic regression.

**Results:**

Gender, indigenous status as well as specific imaging modalities, referral sources and age categories were significantly associated with non‐attendance. Overall, male patients were 1.57 (*P* < 0.001) times more likely to miss a scheduled appointment than female patients. Patients who identified as Aboriginal and Torres Strait Islander were 2.66 (*P* < 0.001) times more likely to miss a scheduled appointment than patients who did not identify as Aboriginal and Torres Strait Islander.

**Conclusions:**

Several key factors appear to affect medical imaging appointment non‐attendance. Key factors include indigenous status, gender, image modality, referral source and age. Further improvement is required to better meet the needs of underrepresented patient demographics.

## Introduction

The identification and reduction of inequalities through social determinants of health remain a global health priority.^1^ Non‐attendance can be considered an indicator of population health inequality, as missed medical appointments have implications for patients’ individual health outcomes and finite healthcare resources.^2^, ^3^

Economic and financial efficiencies in medical imaging are being driven by local, state and federal healthcare targets, and a heightened awareness of the efficient and effective use of high value equipment.^4^ The search for greater efficiency demands greater focus be placed on appropriate patient appointment scheduling.

Booked appointments are commonplace organisational requirements of medical imaging departments. Medical imaging departments provide a vital role in patient care and can provide accurate diagnostic information, reducing the need for investigative surgery or other risky and more costly interventions.^5^, ^6^ Poor attendance rates may have flow on effects in other departments and lead to poorer health and economic outcomes.^7^, ^8^ Therefore, there is value in understanding the rates of non‐attendance in the medical imaging department context and investigating factors that are likely associated.

The rationale for this study was a quality improvement initiative designed to quantify the number of patients who failed to attend their appointment at our imaging department in order to better understand and address factors associated with non‐attendance. In Queensland, the regional (non‐metropolitan) setting accounted for approximately 60% of all public hospital imaging examinations performed.^9^ It has been well documented that Australians living further from metropolitan centres have higher rates of poor health and associated health behaviours with the prevalence greater in the Aboriginal and Torres Strait Islander population.^1^, ^10^, ^11^ Therefore, investigating the factors that are associated with non‐attendance for medical imaging patients in the regional setting is valuable.

Moreover, an analysis of non‐attendance rates is important in order to better identify possible factors and guide tailored strategies to promote attendance. As this was a cross‐sectional cohort analysis, the rates of non‐attendance may vary across departments. However, this snapshot has value in highlighting the significance of non‐attendance rates in the regional clinical context. This will guide service decisions in order to better support patients in taking responsibility for their care.

Studies quantifying non‐attendance rates vary, with figures typically between 6% and 12% but may be much higher in vulnerable subpopulations.^12^, ^13^, ^14^ Demographic factors, such as age, ethnicity and gender, affect the likelihood of non‐attendance in various primary and secondary healthcare settings.^15^, ^16^, ^17^, ^18^

A 2006 case–control study evaluated how patients’ health beliefs impacted non‐attendance at a medical imaging department in the United Kingdom.^19^ The potential for health beliefs such as the perceived importance of disease and associated treatment were found to be less relevant than more practical factors such as choice of appointment and appointment confirmation. This study underlines the value of a quality assurance tool that identifies factors associated with non‐attendance and quantifies these accordingly.

This study aims to identify potential key factors associated with appointment non‐attendance in a public hospital medical imaging department in regional Australia.

## Methods

The research proposal was acknowledged by the HREC chair (HREC/15/QTDD/2) as meeting the requirements of the National Statement of Ethical Conduct in Human Research.^20^ All data were de‐identified and analysed in aggregate, in accordance with the National Statement 5.1.22.

A retrospective audit of all medical imaging outpatient appointments scheduled from 1 January 2015 to 31 December 2015 was performed at a medical imaging department in regional Australia. The department is located in a 250‐bed public hospital facility, which is the largest hospital in the catchment. The medical imaging department employs 23 radiographers, 7 sonographers, 8 registered nurses and 4 radiologists. Appointments for ultrasound, computed tomography (CT), interventional radiology, mammography or a combination of these modalities were included. Other modalities were excluded as they were either not performed in the department at the time of the study or appointments were not routinely scheduled for the modality, such as for general x‐ray examinations. Data were collected using information from an enterprise data reporting system that was already commissioned as part of the department's regular quality assurance activities. The report included fields populated from the hospital information system (HIS) and radiology information system (RIS). These included gender, age, indigenous status, distance from facility, referral source and imaging modality. Each of these fields was considered as possible factors that may be associated with appointment non‐attendance.^2^, ^3^, ^12^, ^14^, ^15^, ^16^, ^17^

For statistical purposes, patients’ ages were grouped into eight subcategories (0–9, 10–17, 18–24, 25–34, 35–44, 45–54, 55–64 and 65 years and over).

The distance category was given three discrete subcategories. Those who lived 50 km or less from the hospital were included in the first subcategory, as the institution defines patients within this distance as not being eligible for travel subsidy. Patients who travelled less than 300 km were generally those within the hospital catchment and are generally able to attend without the need for overnight accommodation. Where the distance travelled was beyond 300 km, this was considered a distance at which patients would likely organise an overnight accommodation.

Referral source was categorised according to the medical specialty of the referrer, with a separate subcategory for patients referred from outside the hospital health service catchment.

The measured outcome was appointment non‐attendance, and dichotomously coded: attended and failed to attend. Failed to attend was defined as those patients who had an outpatient appointment scheduled during the study period and did not arrive for a scheduled appointment and had not made contact indicating that he/she would miss the appointment. The independent variables collected were coded categorically, and were analysed to elucidate the effect that multiple demographics had on the likelihood that a patient would miss a scheduled appointment (Table 1).

**Table 1:** Characteristics of appointments

Factors	*n*	Percentage
Attendance of appointments		
Attended	12,734	94.6
Failed to attend	724	5.4
Gender		
Female	9790	72.7
Male	3669	27.3
Indigenous status		
Not Aboriginal and Torres Strait Islander	12,391	92.1
Aboriginal and Torres Strait Islander	1067	7.9
Age		
0–9 years	265	2.0
10–17 years	199	1.5
18–24 years	1684	12.5
25–34 years	3622	26.9
35–44 years	1591	11.8
45–54 years	1516	11.3
55–64 years	1754	13.0
65 years and over	2827	21.0
Modality		
Modality combination^a^	636	4.7
Ultrasound	9058	67.3
CT^b^	3215	23.9
Interventional radiology	509	3.8
Mammography	40	0.3
Distance from facility		
Under 51 km	9454	70.3
51–300 km	2639	19.6
Over 300 km	1335	9.9
Unknown	30	0.2
Other clinics^c^	904	6.7
Cancer/ONC^d^	1498	11.1
Referral source		
Medical	7664	56.9
Other^e^	1492	11.1
Rural facilities	402	3.0
Surgical	1498	11.1

a Combination of any two or more of the following modalities – ultrasound, CT, mammography, interventional radiology.

b Computed tomography.

c Wards not classed as medical, surgical or palliative.

d Oncology.

e Facilities outside of the regional hospital health service.

Data were analysed using IBM SPSS Statistics v19 (Armonk, NY, USA). Descriptive statistics were used to describe the study population and compare the two groups: those who attended and those who failed to attend. A chi‐squared analysis was used to assess whether an association existed between non‐attendance and demographic factors used in this study. Univariate binary logistic regression was used to calculate the odds ratio (OR) and 95% confidence interval (CI)) for each of the demographic variables associated with not attending their scheduled appointment. Significant univariate variables were then entered in a multivariate (forward) model, and subsequently significant interaction terms between demographic variables remaining in the model were taken into consideration to investigate potential mediating or moderating factors. A *P*‐value of 0.05 was considered statistically significant; all *P*‐values were two‐sided.

## Results

A total of 13,458 appointments were scheduled during the study period, of which 59% were for multiple visits. Each time a patient was scheduled for an appointment they were counted; therefore, each patient could be included more than once.

Table 1 shows characteristics of the appointments. The majority of appointments were for patients aged 18–64 years (75.5%), female (72.7%), non‐Aboriginal and Torres Strait Islander (92.1%) and living within 50 km of the regional facility. The majority of the appointments scheduled were for ultrasound examinations (67.3%) with least (0.3%) appointments scheduled for a diagnostic mammogram.

Overall, patients attended 12,734 (94.6%) booked appointments. Significantly more male patients missed their scheduled appointments than females (7.3% vs. 4.7% respectively, *λ* ^2^(1) = 36.4, *P* < 0.001, Table 2). Of patients identifying as Aboriginal and Torres Strait Islander, 11.8% missed their scheduled appointment. This is compared with (4.8%) of patients who did not identify as Aboriginal and Torres Strait Islander (*λ* ^2^(1) = 93.6, *P* < 0.001). Non‐attendance of scheduled appointment was seen in 7.9% of patients aged 0–9 years, 8.5% of patients 10–17 years and 7.8% of patients 45–54 years. These were significantly higher than the other age categories (*λ* ^2^(7) = 37.54, *P* < 0.001).

**Table 2:** Univariate logistic regression analysis showing likelihood of scheduled appointment non‐attendance by multiple factors

Factors	Proportion of non‐attendance (%)	*P‐*value	Univariate logistic regression		
			Odds ratio (OR)	95% confidence interval (CI)	*P‐*value
Gender					
Female	4.7	<0.001	Reference		
Male	7.3		1.60	1.37–1.87	<0.001
Indigenous status					
Not Aboriginal and Torres Strait Islander	4.8	<0.001	Reference		
Aboriginal and Torres Strait Islander	11.8		2.61	2.13–3.20	<0.001
Age					
0–9 years	7.9	<0.001	1.72	1.06–2.77	0.027
10–17 years	8.5		1.86	1.10–3.15	0.021
18–24 years	4.8		1.01	0.76–1.34	0.958
25–34 years	4.3		0.90	0.71–1.14	0.369
35–44 years	5.7		1.20	0.91–1.57	0.201
45–54 years	7.8		1.68	1.30–2.17	<0.001
55–64 years	6.0		1.28	0.99–1.67	0.062
65 years and over	4.8		Reference		
Modality					
Modality Combination^a^	3.9	<0.001	Reference		
Ultrasound	5.0		1.39	0.91–2.13	0.131
CT^b^	6.5		1.90	1.23–2.95	0.004
Interventional Radiology	7.1		1.91	1.11–3.28	0.020
Mammography	12.5		2.96	0.97–9.02	0.056
Distance from facility					
Under 51 km	5.0	0.02	Reference		
51–300 km	6.4		1.28	1.07–1.54	0.007
Over 300 km	5.9		1.19	0.93–1.52	0.172
Referral source					
Other clinics^c^	3.8	0.006	Reference		
Cancer/ONC^d^	4.9		1.18	0.78–1.78	0.425
Medical	5.2		1.34	0.94–1.89	0.104
Other^e^	5.6		1.31	0.88–1.97	0.188
Rural facilities	7.0		1.81	1.09–3.00	0.023
Surgical	7.1		1.93	1.31–2.84	0.001

a Combination of any two or more of the following modalities – ultrasound, CT, mammography, interventional radiology.

b Computed tomography.

c Wards not classed as medical, surgical or palliative.

d Oncology.

e Facilities outside of the regional hospital health service.

Factors that were significantly associated with non‐attendance in the univariate analysis (Table 2) were entered into a multivariate logistic regression analysis (stepwise forward model) to determine the independent correlates of non‐attendance. Table 3 shows factors significantly associated with non‐attendance in the multivariate model.

**Table 3:** Multivariate logistic regression showing likelihood of scheduled appointment non‐attendance by multiple factors

Factors	Multivariate logistic regression		
	Odds ratio (OR)	95% confidence interval (CI)	*P* value
Gender			
Female	Reference		
Male	1.57	1.31–1.88	<0.001
Indigenous status			
Not Aboriginal and Torres Strait Islander	Reference		
Aboriginal and Torres Strait Islander	2.66	2.15–3.28	<0.001
Age			
0–9 years	1.59	0.96–2.62	0.071
10–17 years	1.67	0.97–2.88	0.065
18–24 years	1.11	0.81–1.53	0.505
25–34 years	1.08	0.82–1.41	0.598
35–44 years	1.30	0.97–1.73	0.080
45–54 years	1.56	1.20–2.02	0.001
55–64 years	1.22	0.94–1.59	0.134
65 years and over	Reference		
Modality			
Modality combination^a^	Reference		
Ultrasound	1.67	1.06–2.63	0.028
CT^b^	1.96	1.24–3.11	0.004
Interventional radiology	1.99	1.14–3.48	0.015
Mammography	3.07	1.00–9.45	0.051
Distance from facility			
<51 km	Reference		
51–300 km	1.14	0.94–1.38	0.176
>300 km	1.00	0.77–1.29	0.990
Referral source			
Other clinics^c^	Reference		
Cancer/ONC^d^	1.23	0.80–1.88	0.347
Medical	1.63	1.14– 2.33	0.007
Other^e^	1.43	0.95–2.16	0.086
Rural facilities	1.94	1.15–3.29	0.014
Surgical	2.34	1.56–3.49	<0.001

a Combination of any two or more of the following modalities – ultrasound, CT, mammography, interventional radiology.

b Computed tomography.

c Wards not classed as medical, surgical or palliative.

d Oncology.

e Facilities outside of the regional hospital health service.

Age was shown to be associated with non‐attendance (*P* < 0.001). From the multivariate logistic regression (Table 3) patient's age at the time of referral showed only those aged between 45 and 54 years were significantly more likely to miss a scheduled appointment (OR 1.56, 95% CI 1.20–2.02) compared with patients 65 years and over. Patients in the 45–54 year age group made up 11.3% of the scheduled appointments and 7.8% of this group failed to attend.

The requested imaging modality was shown to be associated with non‐attendance (*P* < 0.001). The multivariate logistic regression (Table 3) showed patients scheduled for an ultrasound (OR 1.67, 95% CI 1.06–2.63), CT (OR 1.96, 95% CI 1.24–3.11) or interventional radiology (OR 1.99, 95% CI 1.14–3.48) were significantly more likely to miss a scheduled appointment than patients scheduled for a combination of modalities.

Referrals from medical (OR 1.63, 95% CI 1.14–2.32) and surgical (OR 2.34, 95% CI 1.57–3.49) also showed a significant association with patient non‐attendance (Table 3).

Further examination of potential interaction of factors associated with non‐attendance found an interaction between gender and indigenous status (Table 4). When compared to female patients who did not identified as Aboriginal and Torres Strait Islander, male patients who did not identified as Aboriginal and Torres Strait Islander were 1.69 (95% confidence interval [CI] 1.39–2.05) times more likely to miss a scheduled appointment. Female patients who identified as Aboriginal and Torres Strait Islander were 3.06 (95% CI 2.40–3.89) times more likely to miss a scheduled appointment. Male patients who identified as Aboriginal and Torres Strait Islander were 3.00 (95% CI 1.95–4.61) times more likely to miss a scheduled appointment.

**Table 4:** Multivariate logistic regression showing likelihood of scheduled appointment non‐attendance by gender × indigenous status, age, modality, distance from facility and referral source

Factors	Multivariate logistic regression		
	Odds ratio (OR)	95% confidence interval (CI)	*P*‐value
Gender × indigenous status			
Female not Aboriginal and Torres Strait Islander	Reference		
Male not Aboriginal and Torres Strait Islander	1.69	1.39–2.05	<0.001
Female Aboriginal and Torres Strait Islander	3.06	2.40–3.89	<0.001
Male Aboriginal and Torres Strait Islander	3.00	1.95–4.61	<0.001
Age			
0–9 years	1.64	1.00–2.71	0.053
10–17 years	1.68	0.97–2.89	0.063
18–24 years	1.12	0.81–1.53	0.501
25–34 years	1.10	0.83–1.44	0.516
35–44 years	1.32	0.99–1.76	0.061
45–54 years	1.58	1.22–2.05	0.001
55–64 years	1.23	0.94–1.60	0.125
65 years and over	Reference		
Modality			
Modality combination^a^	Reference		
Ultrasound	1.64	1.04–2.59	0.033
CT^b^	1.94	1.22–3.07	0.005
Interventional radiology	1.97	1.13–3.45	0.017
Mammography	3.07	1.00–9.47	0.051
Distance from facility			
<51 km	Reference		
51–300 km	1.14	0.94–1.37	0.194
>300 km	1.00	0.77–1.29	0.978
Referral source			
Other clinics^c^	Reference		
Cancer/ONC^d^	1.23	0.81–1.89	0.335
Medical	1.63	1.14–2.32	0.007
Other^e^	1.45	0.96–2.18	0.079
Rural facilities	1.96	1.16–3.32	0.012
Surgical	2.34	1.57–3.49	<0.001

a Combination of any two or more of the following modalities – ultrasound, CT, mammography, interventional radiology.

b Computed tomography.

c Wards not classed as medical, surgical or palliative.

d Oncology.

e Facilities outside of the regional hospital health service.

## Discussion

In this study of medical imaging department non‐attendance, the rate of non‐attendance was 5.4%, which was similar to that reported by previous publications^21^, ^22^, ^23^, ^24^, ^25^, ^26^ This study appears to be the only analysis of medical imaging non‐attendance in the Australian context, as well as the first to address this subject in the regional setting.

The study has identified a number of factors associated with non‐attendance. Male patients were shown to be 1.57 times less likely to attend their appointment than females. An attitude of self‐reliance and a reluctance to seek help are traits frequently observed in male patients in regional and rural healthcare settings and may partially explain this finding.^27^ This finding agreed with results of most other medical imaging non‐attendance studies.^21^, ^22^, ^24^ However, a Saudi Arabian study of MRI appointment non‐attendance found that females were significantly less likely to attend.^25^ Disparate cultural factors may account for this disagreement.

Patients’ who identified as Aboriginal and Torres Strait Islander were shown to have significantly higher non‐attendance rates. This has been identified as a factor in other studies but this is the first study to investigate this in the medical imaging context.^15^, ^28^ Non‐attendance in this group was 2.66 times higher than that of non‐Indigenous patients. Furthermore, 7.9% of the scheduled appointments in this investigation were for patients who identified as Aboriginal and Torres Strait Islander. The local community has an Aboriginal and Torres Strait Islander population of 4.2%.^29^ Therefore, while Aboriginal and Torres Strait Islanders make up a relatively small section of the community, they are overrepresented in the medical imaging environment and could be considered a vulnerable subpopulation. Greater focus on the cultural capability of clinical staff to improve patient‐centred communication, improved appointment access as well as better access to transport services, may assist in closing the gap in medical imaging appointment attendance rates.^28^, ^30^

While patients aged less than 18 years were less likely to attend than any other age groups, this result was not statistically significant likely due to the small sample size associated. Patients aged 45–54 were the only group shown to be significantly less likely to attend. This result agrees with a recent study of non‐attendance in medical imaging but is at odds with the findings of other medical imaging studies that suggest that patients under 18 or over 65, respectively, are at greater incidence of non‐attendance.^21^, ^22^, ^26^

The main strengths of this study were the large patient cohort allowing for a strong statistical correlation of outcomes. The study highlights the need for medical imaging departments to monitor non‐attendance and consider implementing strategies to mitigate this.

Some study limitations deserve comment. The study was a retrospective audit of data that were collected routinely. Therefore, we were not able to include some of the potential factors as they were not routinely collected, such as day of the week of the appointment.^31^ The available data only contained the date the appointment was made, but not the appointment date itself. Furthermore, some factors, have been found to be relevant to non‐attendance in medical imaging department in other studies such as loud machines, enclosed locations, radiation concerns as well as attitudinal and psychosocial factors; however, they were not collected separately in the existing HIS/RIS databases.^32^, ^33^

To improve compliance and appointment attendance, medical imaging departments should monitor and act in areas where populations are at increased risk of non‐attendance. The barriers and enablers contributing to the identified factors need to be identified through further study.

## Conclusion

This study investigated the immediately available factors associated with appointment non‐attendance at a large regional hospital medical imaging department. Overall, 724 (5.4%) scheduled appointments were missed in the year assessed. Several factors were found to be strongly associated with non‐attendance in the medical imaging department. These included patients that identified as Aboriginal and Torres Strait Islander, male patients, patients aged 45–54 years and patients presenting for particular imaging modalities or referral sources. Further study to identify the specific barriers and enablers for these at‐risk patients will allow medical imaging departments to take steps to further reduce appointment non‐attendance.

## Conflict of Interest

The authors declare no conflict of interest.
